# THBS2 is Closely Related to the Poor Prognosis and Immune Cell Infiltration of Gastric Cancer

**DOI:** 10.3389/fgene.2022.803460

**Published:** 2022-02-03

**Authors:** Shiyu Zhang, Huiying Yang, Xuelian Xiang, Li Liu, Huali Huang, Guodu Tang

**Affiliations:** ^1^ Department of Gastroenterology, The First Affiliated Hospital of Guangxi Medical University, Nanning, China; ^2^ Department of Gastroenterology, The Fifth Affiliated Hospital of Guangxi Medical University, Nanning, China

**Keywords:** gastric cancer, biomarker, immune infiltration, prognostic index, bioinformatics

## Abstract

**Background:** The potential functions of Thrombospondin 2 (THBS2) in the progression and immune infiltration of gastric cancer (GC) remain unclear. The purpose of this study was to clarify the role of THBS2 in GC prognosis and the relationship between THBS2 and GC immune cell infiltration.

**Material and Methods:** The differential expression levels of THBS2 in the GC and cancer-adjacent tissues were identified using the TCGA databases and verified using real-time polymerase chain reaction (PCR), immunohistochemical staining and two datasets from Gene Expression Omnibus (GEO). THBS2 related differential expressed genes (DEGs) were identified and used for further functional enrichment analysis and Gene Set Enrichment Analysis (GSEA). Furthermore, a THBS2-related immune infiltration analysis was also performed. Kaplan-Meier and Cox regression analyses were utilized to illustrate the effects of THBS2 on the prognosis and clinical variables of GC. Finally, a nomogram was constructed to predict the survival probability of patients with GC.

**Results:** The THBS2 expression in GC was significantly higher than that in cancer-adjacent tissues (*p* < 0.001), which was verified using real-time PCR, immunohistochemical staining and datasets from GEO. The 599 identified DEGs were primarily enriched in pathways related to tumorigenesis and tumor progression, including the focal adhesion pathway, signaling by vascular endothelial growth factor, and Wnt signaling. THBS2 expression was positively correlated with the enrichment of the macrophages (r = 0.590, *p* < 0.001), which was also confirmed by immunohistochemistry; however, negatively correlated with the enrichment of Th17 cells (r = 0.260, *p* < 0.001). The high expression of THBS2 was significantly correlated with the pathological grade (*p* < 0.01), histological grade (*p* < 0.05), histological type (*p* < 0.05), T stage (*p* < 0.001), and poor overall survival (OS) (*P =* 0.003) of GC. The constructed nomogram can well predict the 1-, 3-, and 5-years OS probability of patients with GC (C-index [95% confidence interval] = 0.725 [0.701–0.750]).

**Conclusion:** THBS2 is closely related to the poor prognosis and immune infiltration of gastric cancer.

## Introduction

Gastric cancer (GC) is the fifth most common malignancy and the third most common cause of death globally (Park et al., 2018). Currently, the treatment of GC includes surgery (Herrera-Almario and Strong, 2016), radiotherapy (Tey et al., 2017), neoadjuvant chemotherapy (Zhandossov et al., 2018), and immunotherapy (Niccolai et al., 2015). Due to the improvement in endoscopic screening technology, in countries with a high incidence of GC, such as Japan and South Korea, more than 50 percent of GC patients can be diagnosed at an early stage (So et al., 2021). However, in most countries with poor screening technology, most patients are diagnosed at an advanced GC stage, which leads to poor treatment outcomes (Choi et al., 2015; Inokuchi et al., 2018). Therefore, biomarkers related to the progression and GC prognosis should be explored to improve the therapeutic effect and reduce mortality owing to GC.

Thrombospondin-2 (THBS2) is a member of the matricellular calcium-binding glycoprotein family, which interacts with growth factors, cell receptors, and extracellular matrix (ECM), and THBS2 plays an important role in cell proliferation, adhesion and apoptosis (Bornstein et al., 2000; Zhuo et al., 2016). It has also been reported that THBS2 may be a serum biomarker in the diagnosis of colon cancer (Wang et al., 2016) and lung cancer (Weng et al., 2016). Bioinformatics analysis suggested that THBS2 might be a potential biomarker for GC (Cao et al., 2018). An *in vitro* study showed that THBS2 silencing inhibited the proliferation, migration and invasion of gastric cancer cells (Ao et al., 2018). The results of Chuanjun Zhuo showed that GC patients with low THBS2 expression had a better prognosis (Zhuo et al., 2016). However, there have also been contrasting reports. It has been reported that the expression of THBS2 is down-regulated in most GC patients, and the higher the expression of THBS2, the better the prognosis of GC patients, but the sample size was too small, only 14 cases (Sun et al., 2014). Therefore, the role of THBS2 in the prognosis of GC needs to be verified further.

Since immune checkpoint blocking therapy is used to treat several types of tumors, some patients have achieved significant clinical responses (Ribas and Wolchok, 2018). In September 2017, pembrolizumab (anti-programmed death 1 [PD1] antibody) was approved to treat GC or gastroesophageal junction cancer; however, the response rate was relatively low (Fashoyin-Aje et al., 2019). The effectiveness of tumor immunotherapy should be based on the premise that effector cells infiltrate into the tumor microenvironment (TME) (Kirkwood et al., 2012). Anti-ctla-4 (T lymphocyte associated antigen 4) monoclonal antibody plays an antitumor role by prolonging T cell stimulation and restoring T cell proliferation (Engelhardt et al., 2006). T cells are the only type of tumor-infiltrating lymphocytes (TILs), and other common TILs cells include macrophages and NK cells. Therefore, understanding the tumor microenvironment and TILs of GC may lay a certain foundation for improving the effect of immunotherapy in GC. However, it has not been reported whether THBS2 can affect the TILs abundance of GC.

In this study, we analyzed the significance of THBS2 in GC in the TCGA database and Gene Expression Omnibus (GEO) database using bioinformatics analysis, including differential expressed genes (DEGs) analysis, functional enrichment analysis, gene set enrichment analysis (GSEA), immune cell infiltration analysis, clinical correlation analysis, and survival analysis. Moreover, a nomogram was also created to predict the overall survival (OS) of patients with GC.

## Materials and Methods

### Data Sources

We downloaded gene expression data and clinical information for GC, which included 32 cancer-adjacent tissues and 375 tumor tissues, from TCGA (https://portal.gdc.cancer.gov/, accessed time: July 31, 2021). Samples with incomplete clinical data were excluded. RNAseq data was converted by log2 in R software for subsequent analysis. The clinical characteristic of GC is presented in Table 1. Additionally, two microarray datasets (GSE54129 and GSE13911) containing GC and cancer-adjacent tissues were downloaded from GEO online database (https://www.ncbi.nlm.nih.gov/geo/, accessed time: December 17, 2021).

**TABLE 1:** The clinical characteristic of STAD.

Characteristic	Levels	Overall
n		375
Gender, n (%)	Female	134 (35.7%)
	Male	241 (64.3%)
Age, n (%)	≤ 65	164 (44.2%)
	>65	207 (55.8%)
T stage, n (%)	T1	19 (5.2%)
	T2	80 (21.8%)
	T3	168 (45.8%)
	T4	100 (27.2%)
N stage, n (%)	N0	111 (31.1%)
	N1	97 (27.2%)
	N2	75 (21%)
	N3	74 (20.7%)
M stage, n (%)	M0	330 (93%)
	M1	25 (7%)
Histological type, n (%)	Diffuse Type	63 (16.8%)
	Mucinous Type	19 (5.1%)
	Not Otherwise Specified	207 (55.3%)
	Papillary Type	5 (1.3%)
	Signet Ring Type	11 (2.9%)
	Tubular Type	69 (18.4%)
Pathologic stage, n (%)	Stage I	53 (15.1%)
	Stage II	111 (31.5%)
	Stage III	150 (42.6%)
	Stage IV	38 (10.8%)
Histologic grade, n (%)	G1	10 (2.7%)
	G2	137 (37.4%)
	G3	219 (59.8%)
Residual tumor, n (%)	R0	298 (90.6%)
	R1	15 (4.6%)
	R2	16 (4.9%)
Primary therapy outcome, n (%)	PD	65 (20.5%)
	SD	17 (5.4%)
	PR	4 (1.3%)
	CR	231 (72.9%)
*H pylori* infection, n (%)	No	145 (89%)
	Yes	18 (11%)
Barrett’s esophagus, n (%)	No	193 (92.8%)
	Yes	15 (7.2%)
Anatomic neoplasm subdivision, n (%)	Antrum/Distal	138 (38.2%)
	Cardia/Proximal	48 (13.3%)
	Fundus/Body	130 (36%)
	Gastroesophageal Junction	41 (11.4%)
	Other	4 (1.1%)
Age, median (IQR)		67 (58, 73)

### THBS2 *Differential Expression in Pan-Cancer and GC Tissues*

Log2-converted TPM RNAseq data for cancer-adjacent and tumor tissues (TCGA) were obtained from the UCSC XENA. The differential expression between tumor and non-tumor tissues was tested using Wilcoxon Rank Sum Test and visualized through the box and scatter plots. Additionally, the receiver operating characteristic (ROC) curve was constructed to assess the predictive diagnostic value of THBS2 expression.

### Real-Time PCR of THBS2 Expressions in GC and Adjacent Tissues

This study has been approved by the Ethics Committee of the First Affiliated Hospital of Guangxi Medical University and informed consent of all patients. From May to July 2021, cancer and para-cancerous biopsy specimens were incessantly collected from 16 patients diagnosed with GC at the Endoscopy Center of the First Affiliated Hospital of Guangxi Medical University (Nanning, Guangxi). All tissue specimens were immersed in RNA protective solution and quickly transferred to a –80°C refrigerator for preservation. All patients had not received any prior treatment for the tumor, including radiation or chemotherapy. Furthermore, patients who were complicated with other known tumors were excluded. The clinical information of these 16 patients is presented in Table 2.

**TABLE 2:** Clinical information of 16 patients with gastric cancer for Real-time PCR.

Gender (M/F)	Age (years)	Histological type	Histological grade	Anatomic neoplasm subdivision
M	76	Signet Ring Type	G3	Antrum
M	54	Tubular Type	G3	Antrum
M	73	Tubular Type	G1	Antrum
F	59	Mucinous Type	G3	Fundus/Body
M	49	Papillary Type	G3	Antrum
F	37	Signet Ring Type	G3	Antrum
M	60	Tubular Type	G2	Antrum
M	76	Tubular Type	G1	Antrum
F	48	Signet Ring Type	G3	Cardia
M	65	Papillary Type	G2	Cardia
M	70	Diffuse Type	G3	Antrum
M	82	Tubular Type	G1	Fundus/Body
M	67	Diffuse Type	G2	Cardia
M	67	Tubular Type	G2	Cardia
F	77	Tubular Type	G2	Cardia
F	52	Diffuse Type	G3	Antrum

### RNA Extraction and Quantitative Real-Time PCR

First, total RNA from tissues was extracted using the Trizol reagent (R0016, Beyotime). Then, the first-strand cDNA was synthesized from the total RNA via the PrimeScript™ RT Reagent Kit with gDNA Eraser (RR047A, Takara). After that, the expression of the THBS2 gene was normalized to GAPDH expression. The expression level of the THBS2 gene was computed by using the 2^–∆∆^ Ct way.

Specific primer base sequence:

THBS2-F: 5′-ATC​ACA​CGC​ATC​CGT​CTC​TG-3’.
THBS2-R: 5′-ATC​ACA​CGC​ATC​CGT​CTC​TG-3’.
GAPDH-F: 5′-GTC​AGC​CGC​ATC​TTC​TTT-3’.
GAPDH-R: 5′-CGC​CCA​ATA​CGA​CCA​AAT-3’.

### Immunohistochemistry

We continuously collected tumor and para-tumor tissues from 80 GC patients without other known malignant tumors after surgery in Suqian First People’s Hospital from January 2017 to April 2019. None had a history of chemotherapy or radiation therapy before surgery. All the tissues were modified into tissue chips for further IHC staining. Dewaxing, and hydration were performed before the THBS2 primary antibody (ab112543, Abcam, 1:1000) was added and incubated at 4°C for 12 h. After incubating with HRP labeled linked polymer (KIT-5009, MXB biotechnologies) at 26°C for 40 min, signal detection was performed using DAB (P0202, Beyotime). The expression of THBS2 was measured using ImageJ software and visualized with GraphPad Prism 8.0 after statistical analysis.

### Identification of DEGs Between High and low Expression Groups of THBS2

According to the mean value of THBS2 expression, all the samples from TCGA datasets were divided into high and low expression groups. RNA-Seq expression data were processed using the DESeq2 package, and DEGs were defined with a p. adj <0.05 and an absolute logFC >1.5. Detailed gene expression is shown in the volcano map.

### Functional Enrichment Analysis of DEGs

In this study, or. Hs.eg.db package was used for ID conversion, while the clusterProfiler package was used for enrichment analysis. The threshold conditions included: p. adj <0.05, and q value < 0.2. Statistical analysis and GSEA visualization are performed by the clusterProfiler package, using C2 collection from MSigDB. Permutations with 10,000 times were performed by gene set, and significance was set as an adjusted *P* < 0.05 and false discovery rate (FDR) < 0.25.

### Immune Cell Infiltration

The relative tumor infiltration levels of immune cell types were quantified using ssGSEA of GSVA package (Hänzelmann et al., 2013) to interrogate gene expression levels in published signature gene lists (Bindea et al., 2013). To explore the correlation between THBS2 and the immune infiltration levels and the association of immune infiltration with the different expression groups of THBS2, Spearman’s correlation and Wilcoxon signed-rank sum test was adopted.

Furthermore, the average optical density (AOD) of THBS2 expression was calculated using ImageJ software based on immunohistochemistry results of 80 GC patients. According to the mean value of AOD of THBS2, all 80 GC patients were divided into high and low expression groups. Finally, 10 cases were randomly selected from the high expression and low expression groups for immunohistochemical staining of macrophages and NK cells. The immunohistochemical procedure was the same as part 2.5, and we used CD56 (MAB-0743, MXB biotechnologies) and CD68 (MAB-0041, MXB biotechnologies) to label NK cells and macrophages, respectively. The proportion of CD56/CD68 positive area was measured using ImageJ software and visualized with GraphPad Prism 8.0 after statistical analysis.

### Clinical Correlation Analysis of THBS2 in GC

Wilcoxon signed-rank sum test and logistic regression were used to evaluate the correlation between THBS2 expression and clinicopathological variables. Further, univariate and multivariate Cox regressions were used to compare the effect of THBS2 expression and other clinicopathological variables on survival in patients with GC. Independent factors for GC prognosis were identified using multivariate Cox regression analysis.

Furthermore, we collected clinicopathological data from 80 patients who underwent THBS2 immunohistochemistry to evaluate the relationship between THBS2 expression and clinicopathological variables. Chi-square tests were used to evaluate the relationship between gender, pathological type, residual tumor status, and THBS2 expression. Fisher’s exact tests were used to evaluate the relationship between pathologic stage, T stage, N stage, primary treatment outcome, and THBS2 expression. Wilcoxon signed Rank-Sum test was used to evaluate the relationship between age and THBS2 expression.

### Construction and Verification of Nomogram

A nomogram used to predict the probability of survival at 1, 3, and 5 years for patients with GC included all independent prognostic factors. The prognostic data were derived from published studies (Liu et al., 2018). Nomogram was formulated using R with the survival and rms package. We used the bootstrap method to calculate the parameters, and the number of repeated calculations for each group of samples was 200. The C-index is used to evaluate the prediction ability of the nomogram; the closer C-index is to 1, the stronger the prediction ability is.

## Results

### THBS2 Differential Expression in Pan-Cancer and GC Tissues

**FIGURE 1:**
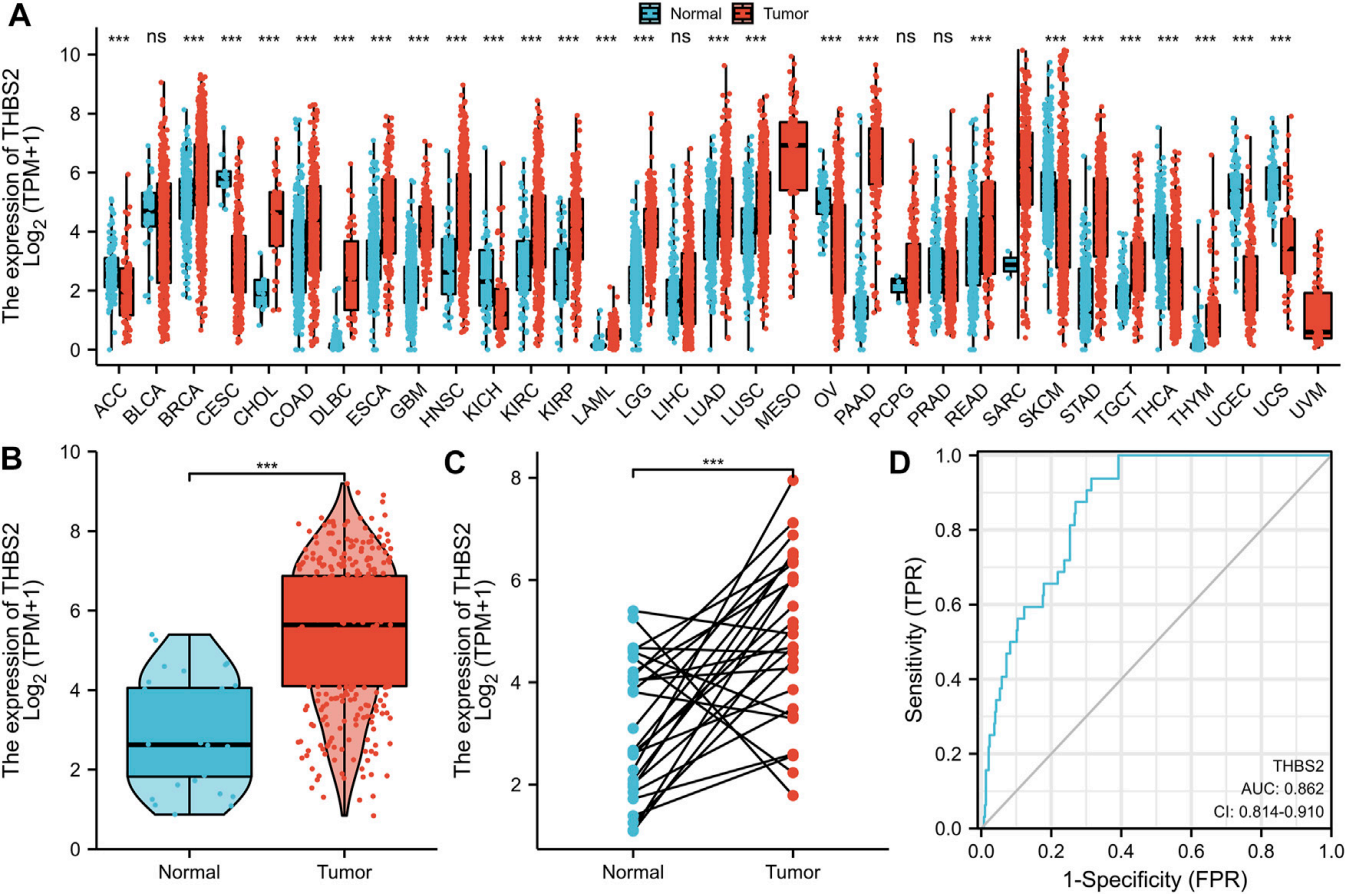
Differential expression of THBS2 in different tumors and THBS2-related differentially expressed genes (DEGs). **(A)** Differential expression of THBS2 of different cancers compared with cancer-adjacent tissues in the TCGA database. **(B,C)** Differential expression of THBS2 in GC. **(D)** ROC curve was used to calculate the predictive diagnostic value of THBS2 expression between GC and para-cancer tissues. Significance marker: NS, *p* ≥ 0.05; *, *p* < 0.05; **, *p* < 0.01; ***, *p* < 0.001.

Significant differential expression was observed in most of the 33 cancers, as illustrated in Figure 1A. Secondly, the THBS2 expression in 375 STAD samples and 32 para-cancer samples in the TCGA was compared. There was a significant difference in THBS2 expression between GC tissues and cancer-adjacent tissues (*P* < 0.001) (Figure 1B). The THBS2 expression in GC tissues was also significantly higher than that in the corresponding adjacent tissues (*P* < 0.001) (Figure 1C).

The area under the curve (AUC) of the ROC curve was used to evaluate the predictive value of THBS2 for the diagnosis of GC. The result of the ROC curve (Figure 1D) demonstrated that THBS2 had a high predictive value in distinguishing GC from cancer-adjacent tissues (AUC = 0.864, 95%CI: 0.812–0.915).

**FIGURE 2:**
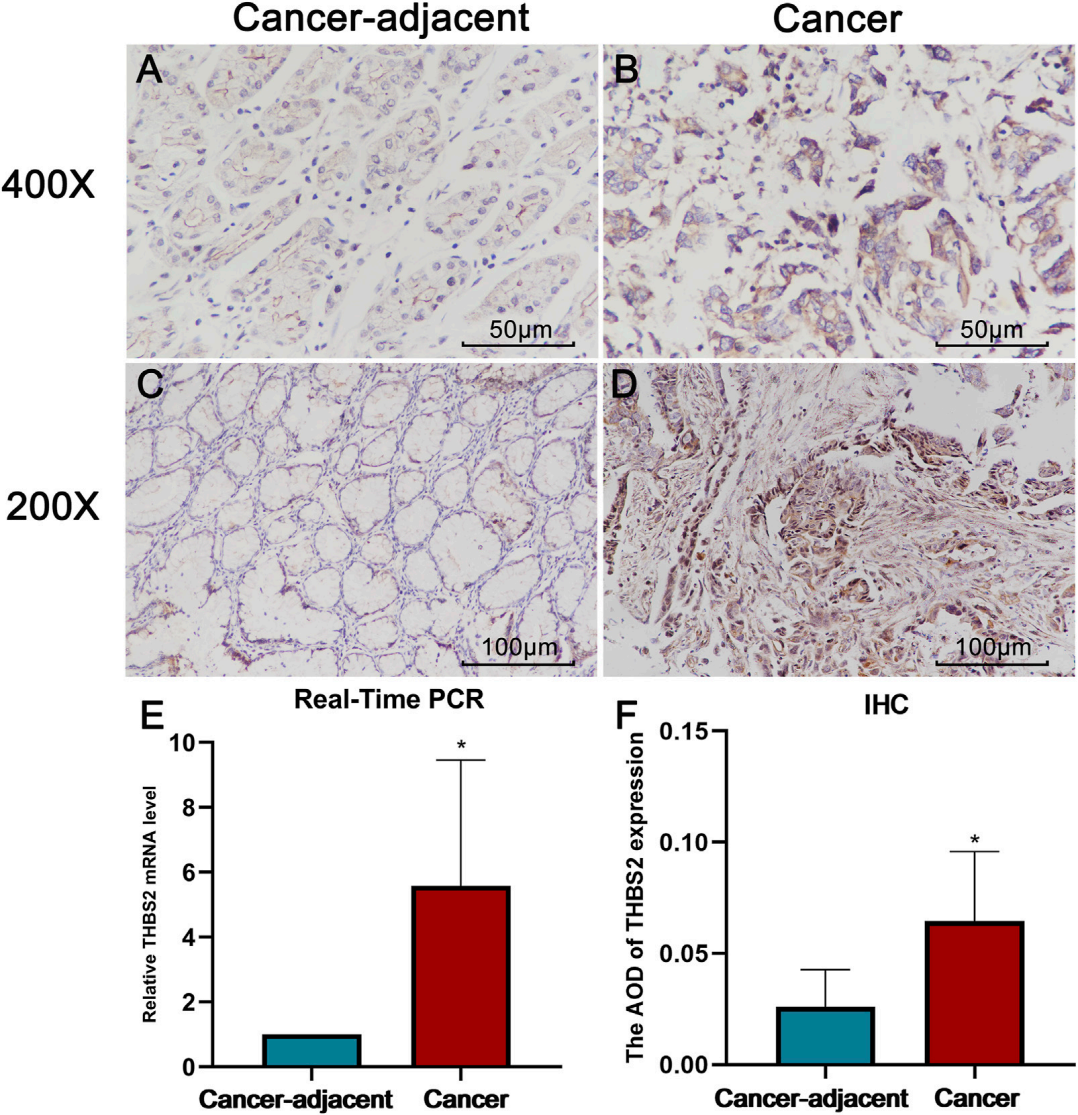
The results of Real-Time PCR and Immunohistochemistry (IHC). **(A)** THBS2 expression in cancer-adjacent tissue (400X). **(B)** THBS2 expression in GC tissue (400X). **(C)** THBS2 expression in cancer-adjacent tissue (200X). **(D)** THBS2 expression in GC tissue (200X). **(E)** Relative THBS2 mRNA level in cancer-adjacent and GC tissues. GC: Gastric cancer. *, *p* < 0.05.

The real-time PCR results further verified the reliability of our differential analysis at the transcriptional level (Figure 2E Cancer-adjacent vs. Cancer, *P =* 0.021). Moreover, the same results were also observed in IHC (Figure 2F, Cancer-adjacent vs. Cancer, *P =* 0.010).

**FIGURE 3:**
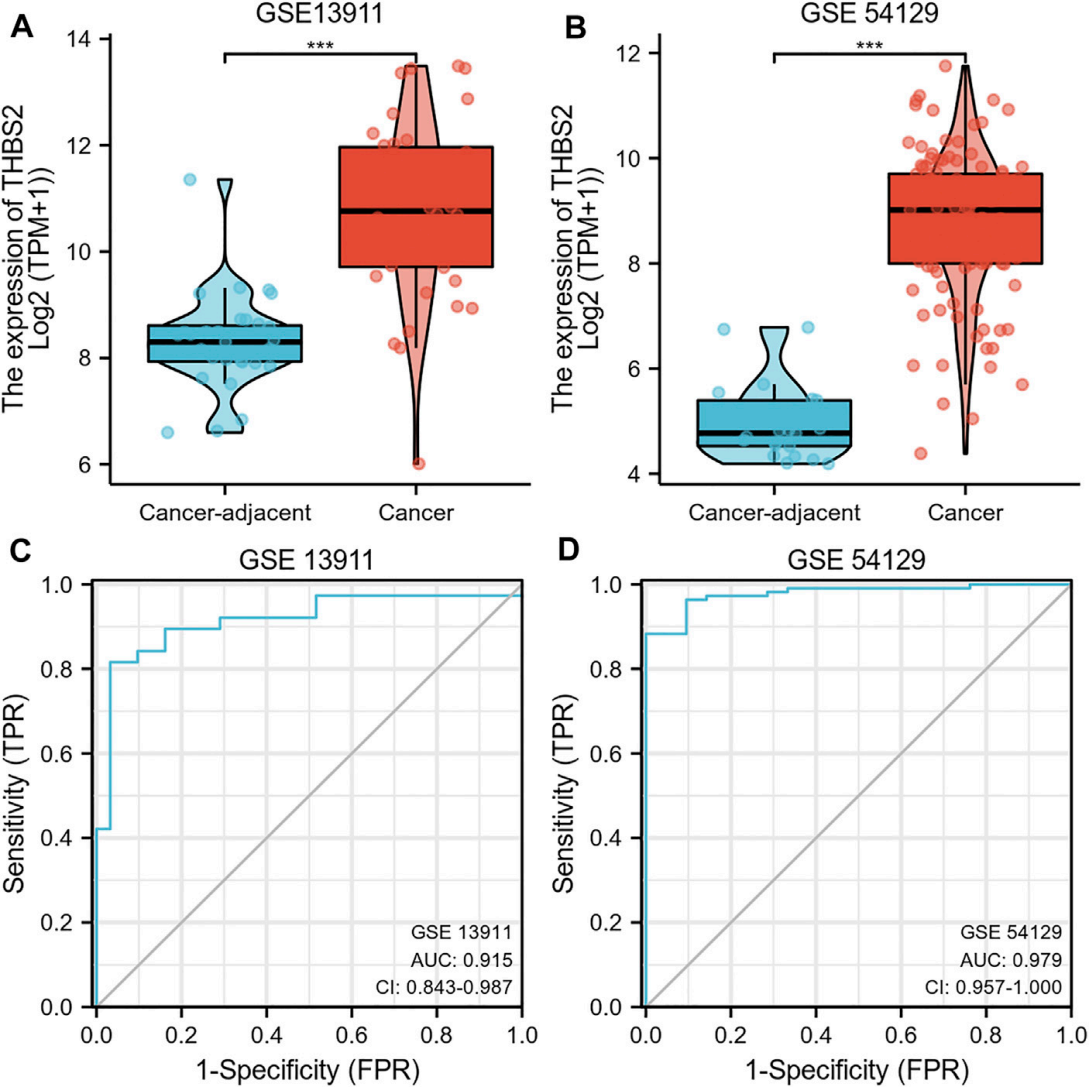
**(A)** Differential THBS2 mRNA expression between GC and cancer-adjacent tissues in GSE13911. **(B)** Differential THBS2 mRNA expression between GC and cancer-adjacent tissues in GSE54129. **(C)** ROC curve used to calculate the predictive diagnostic value of THBS2 expression between GC and para-cancer tissues (GSE 13911). **(D)** ROC curve used to calculate the predictive diagnostic value of THBS2 expression between GC and para-cancer tissues (GSE54129). TPM: Transcripts Per Kilobase of exon model per Million mapped reads. GC, Gastric cancer; Significance marker: ***, *p* < 0.001.

We further analyzed THBS2 mRNA expression in GC and cancer-adjacent tissues RNA sequencing data from GSE13911 and GSE54129. The results showed that the THBS2 mRNA expression level was higher in GC than in cancer-adjacent tissues (Figure 3A, B, *p <* 0.001). The result of the ROC curve demonstrated that THBS2 had a high predictive value in distinguishing GC from cancer-adjacent tissues (Figure 3C, GSE13911, AUC = 0.915, 95%CI: 0.843–0.987; Figure 3D, GSE54129, AUC = 0.979, 95%CI: 0.957–1.000).

### DEGs Identification and Functional Enrichment Analysis of DEGs

**FIGURE 4:**
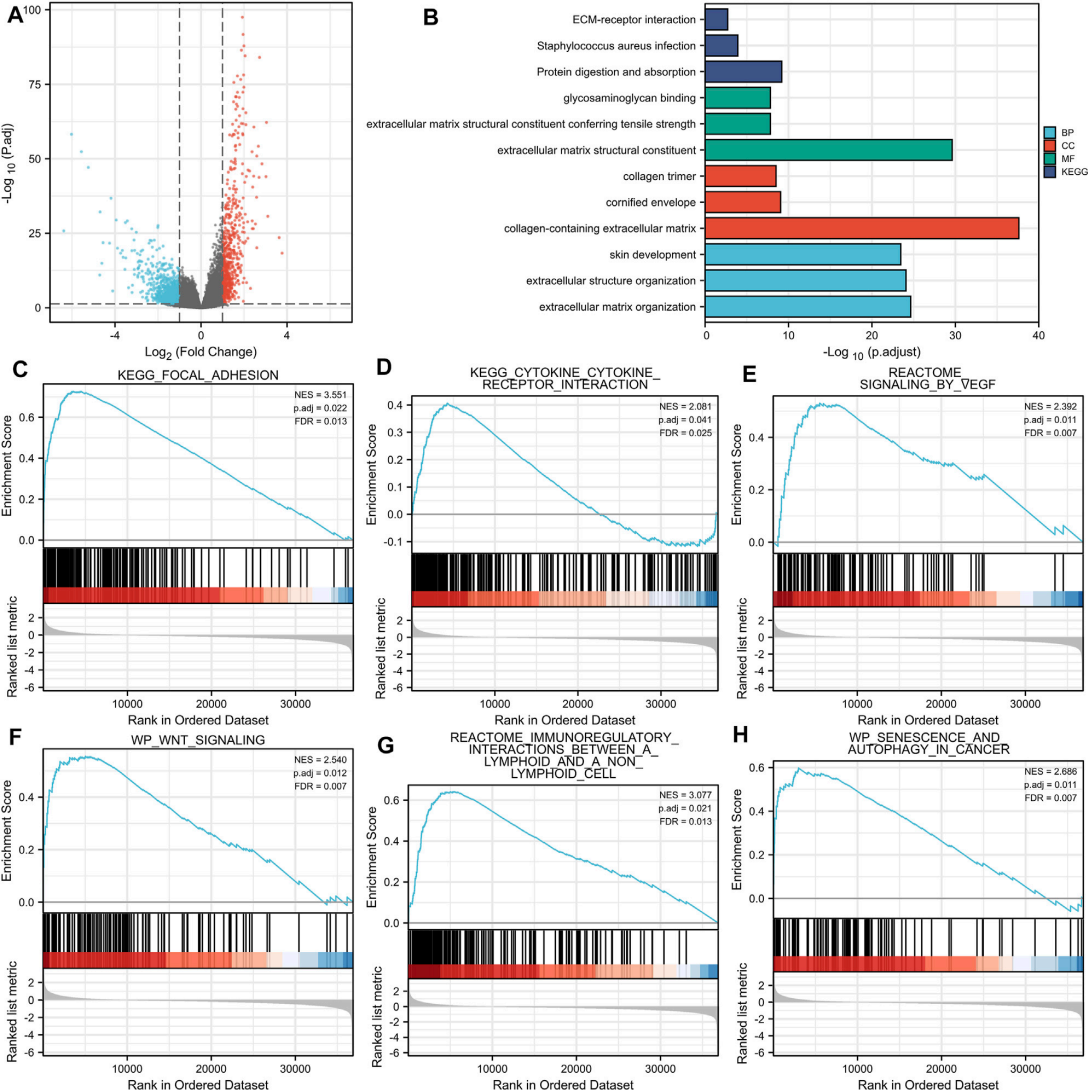
**(A)** Volcano plots of the DEGs. Blue represents down-regulated DEGs, red represents up-regulated DEGs. **(B)**: The top three items enriched in biological processes (BP), cellular component (CC), molecular function (MF), and Kyoto Encyclopedia of Genes and Genomes (KEGG) of DEGs. **(C–H)**: Enrichment plots from the gene set enrichment analysis (GSEA). NES, normalized enrichment score; p. adj, adjusted *p* value; FDR, false discovery rate.

A total of 599 DEGs were identified, including 170 up-regulated and 429 down-regulated DEGs (Figure 4A).

Enriched biological processes (BP), cellular components (CC), and molecular function (MF) were used to comprehend the biological functions of DEGs better. The top three items enriched in BP, CC, MF, and KEGG of DEGs were visualized. As shown in Figure 4B, THBS2-related genes were involved in KEGG (Protein digestion and absorption, Staphylococcus aureus infection, and ECM-receptor interaction); MF (extracellular matrix structural constituent, extracellular matrix structural constituent conferring tensile strength, and glycosaminoglycan binding); CC (collagen-containing extracellular matrix, cornified envelope, and collagen trimer); and BP (extracellular matrix organization, extracellular structure organization, and skin development). The details of GO and KEGG enrichment analysis results are illustrated in Table 3.

**TABLE 3:** Details of GO and KEGG enrichment analyses.

Ontology	ID	Description	Gene ratio	Bg ratio	P Value	p.adjust	q value
BP	GO:0030198	extracellular matrix organization	49/329	368/18,670	7.35e-29	2.25e-25	1.97e-25
BP	GO:0043062	extracellular structure organization	51/329	422/18,670	5.45e-28	8.36e-25	7.30e-25
BP	GO:0043588	skin development	50/329	419/18,670	3.46e-27	3.53e-24	3.09e-24
BP	GO:0070268	cornification	29/329	112/18,670	6.02e-26	4.62e-23	4.03e-23
BP	GO:0030216	keratinocyte differentiation	41/329	305/18,670	2.15e-24	1.32e-21	1.15e-21
CC	GO:0062023	collagen-containing extracellular matrix	63/352	406/19,717	9.53e-41	2.47e-38	2.24e-38
CC	GO:0001533	cornified envelope	14/352	65/19,717	6.92e-12	8.96e-10	8.12e-10
CC	GO:0005581	collagen trimer	15/352	87/19,717	3.64e-11	3.15e-09	2.85e-09
CC	GO:0044420	extracellular matrix component	12/352	51/19,717	7.39e-11	4.78e-09	4.34e-09
CC	GO:0005583	fibrillar collagen trimer	7/352	11/19,717	1.69e-10	7.30e-09	6.61e-09
MF	GO:0005201	extracellular matrix structural constituent	39/316	163/17,697	5.72e-33	2.43e-30	2.06e-30
MF	GO:0030020	extracellular matrix structural constituent conferring tensile strength	11/316	41/17,697	9.70e-11	1.50e-08	1.27e-08
MF	GO:0005539	glycosaminoglycan binding	22/316	229/17,697	1.54e-10	1.50e-08	1.27e-08
MF	GO:0005518	collagen binding	13/316	67/17,697	1.57e-10	1.50e-08	1.27e-08
MF	GO:0048018	receptor ligand activity	32/316	482/17,697	1.77e-10	1.50e-08	1.27e-08
KEGG	hsa04974	Protein digestion and absorption	17/147	103/8,076	3.14e-12	6.47e-10	5.92e-10
KEGG	hsa05150	Staphylococcus aureus infection	11/147	96/8,076	1.17e-06	1.21e-04	1.10e-04
KEGG	hsa04512	ECM-receptor interaction	9/147	88/8,076	2.89e-05	0.002	0.002
KEGG	hsa04972	Pancreatic secretion	9/147	102/8,076	9.32e-05	0.004	0.004
KEGG	hsa05146	Amoebiasis	9/147	102/8,076	9.32e-05	0.004	0.004

GSEA analysis revealed that THBS2-related enrichment pathways were as follows: focal-adhesion, vascular endothelial growth factor (VEGF) signaling, Wnt signaling, immunoregulatory lymphoid, and a non-lymphoid cell, senescence, and autophagy in cancer (Figures 4C–H).

### THBS2 Expression is Closely Related to the Immune cell Infiltration and Clinicopathological Variables of Gastric Cancer

**FIGURE 5:**
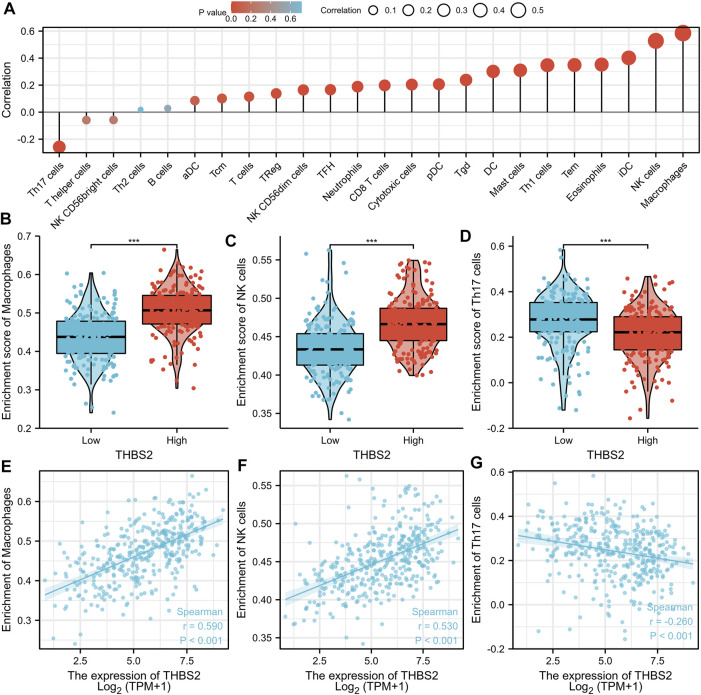
The Correlation Between THBS2 Expression and Immune Infiltration. **(A)** Correlation between the relative abundances of immune cells and THBS2 expression level. The size of dots is positively related to the absolute value of Spearman R. **(B–D)** The difference of immune cells (Macrophages, NK cells, and Th17 cells) between the high and low expression groups based on the median value of THBS2 expression. **(E–G)** The correlation of immune cells (Macrophages, NK cells, and Th17 cells) between the high and low expression groups based on median value of THBS2 expression.

**FIGURE 6:**
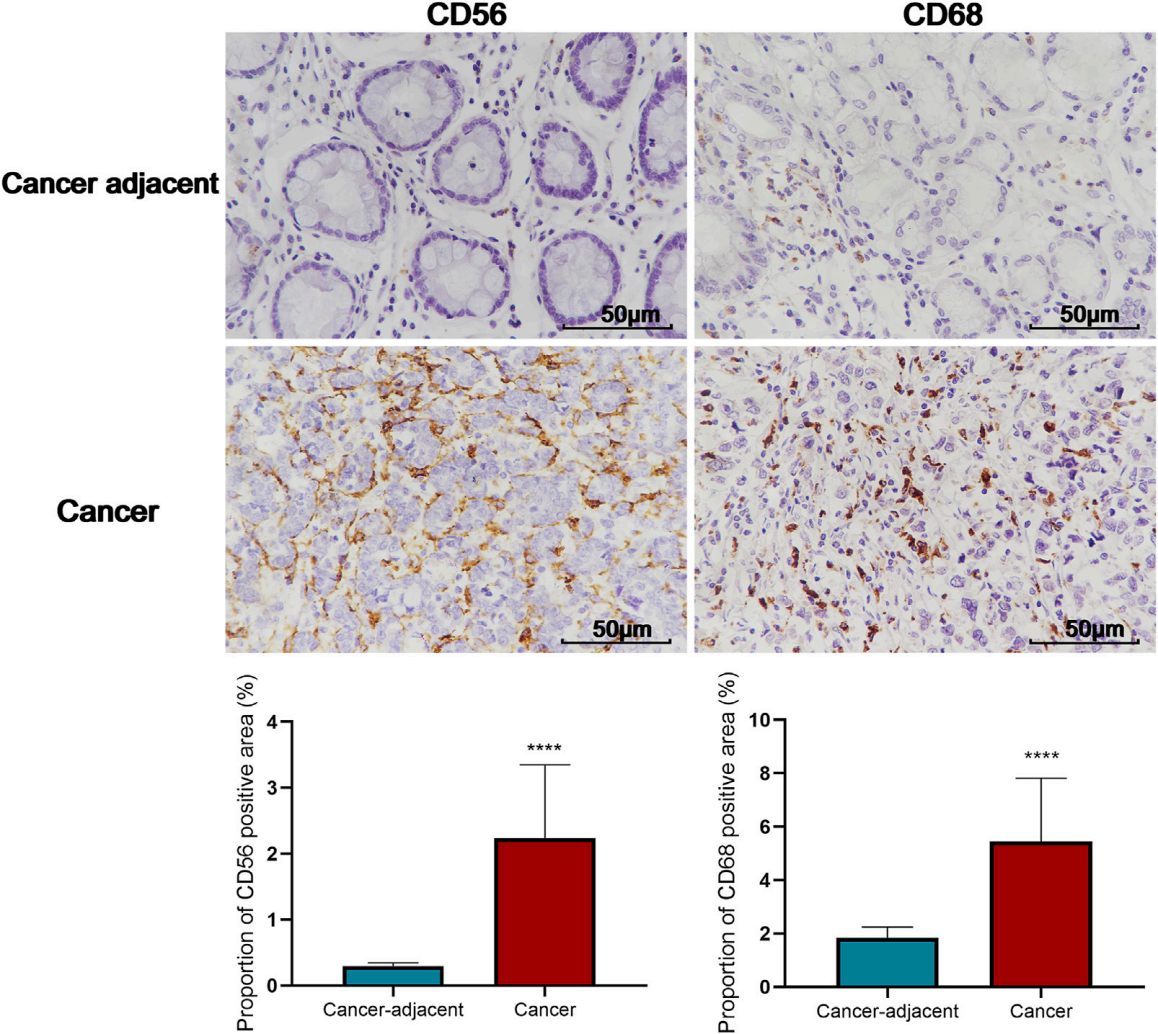
Comparison of the proportion of CD56 and CD68 positive area in tumor and cancer-adjacent tissues (400X). C-D: Comparison of the proportion of CD56 and CD68 positive area in THBS2 high and low expression groups; E-F: Correlation between THBS2 expression and the proportion of CD56/CD68 positive area. ****, *p* < 0.0001.

**FIGURE 7:**
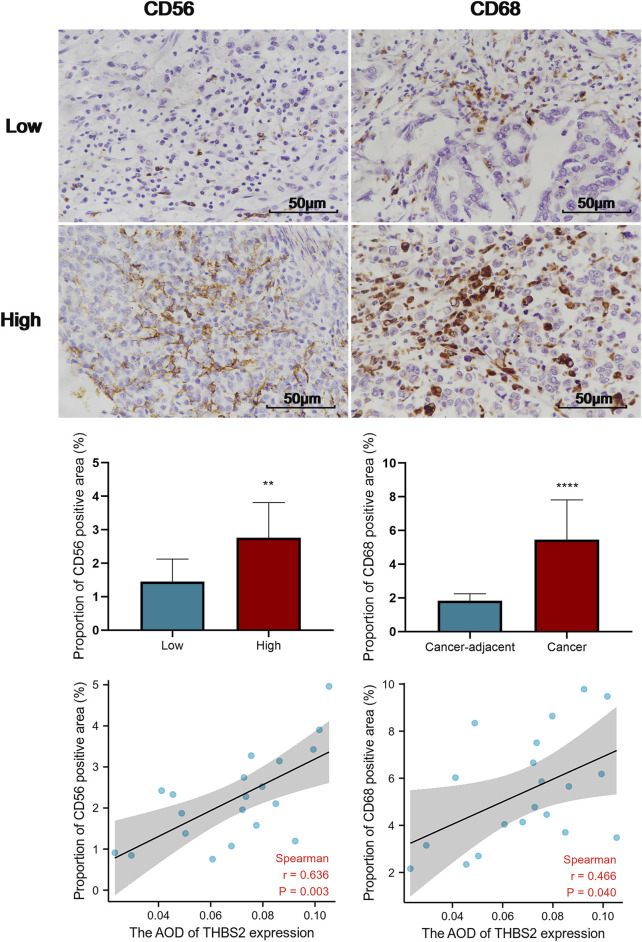
Comparison of the proportion of CD56 and CD68 positive area in THBS2 high and low expression groups, and the correlation between THBS2 expression and the proportion of CD56/CD68 positive area (400X). AOD, average optical density; **, *p* < 0.01; ****, *p* < 0.0001.

An analysis based on online data showed that the higher the expression of THBS2, the more macrophages, NK cells, iDC, and eosinophils infiltrate, and the fewer Th17 cells, T helper cells, and NK CD56 bright cells infiltrate in GC tissues. (Figures 5A–G). We further verified the differential enrichment of macrophages and NK cells in the tumor and cancer-adjacent tissues by immunohistochemistry. The proportion of positive area of CD56 (NK cells) and CD68 (macrophages) in GC tissues was significantly higher than that in cancer-adjacent tissues (Figure 6; CD56, Cancer vs. Cancer-adjacent, *P* < 0.0001; CD68, Cancer vs. Cancer-adjacent, *P* < 0.0001). The proportion of the positive area of CD56 and CD68 in the THBS2 high expression group was significantly higher than that in the low expression group (Figure 7; CD56, High vs. Low, *P* < 0.01; CD68, High vs. Low, *P* < 0.0001). Finally, we verified that the proportion of CD56 and CD68 positive area was significantly correlated with THBS2 expression (Figure 7; CD56, r = 0.636, *P* < 0.01; CD68, r = 0.466, *P* < 0.05).

**FIGURE 8:**
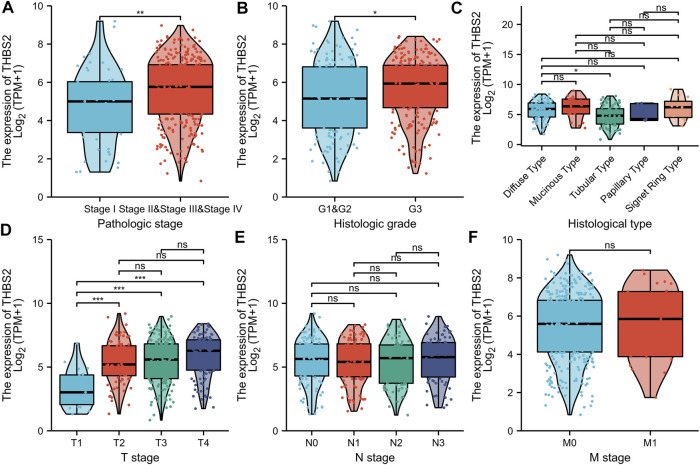
Association with THBS2 expression and clinicopathological characteristics. **(A)** pathologic stage, **(B)** histological grade, **(C)** histologic type, **(D)** T stage **(E)** N stage, and **(F)** M stage in GC patients in TCGA cohort. TCGA, The Cancer Genome Atlas; GC, gastric cancer.

There were 375 patients with complete clinicopathologic data in the TCGA database who were included in the cohort. As illustrated in Figures 8A–D, the high expression of THBS2 was significantly correlated with the pathological grade (stage I vs. stage II and III & IV, *P* < 0.01), histological grade (G1 & G2 vs. G3, *P* < 0.05), histological type (Diffuse Type vs. Tubular Type, *P* < 0.05), and T stage (T1 vs. T2, T1 vs. T3, T1 vs. T4, *P* < 0.001) of patients. Moreover, there was no significant statistical correlation between the THBS2 expression level and N and M stages (Figures 8E,F).

We further analyzed the relationship between THBS2 expression and clinicopathological data of 80 patients who underwent THBS2 immunohistochemistry. The results showed that THBS2 expression was significantly correlated with pathologic stage (*P* = 0.044), T stage (P = 0.003), histologic type (*p* < 0.001), and histological grade (*P* = 0.027) in 80 GC patients who underwent THBS2 immunohistochemistry (Table 4).

**TABLE 4:** The relationship between THBS2 expression and clinicopathological variables in 80 patients underwent immunohistochemistry.

Characteristic	Low	High	p
n	38	42	
Gender (M/F), n (%)			0.634
F	9 (11.2%)	13 (16.2%)	
M	29 (36.2%)	29 (36.2%)	
Pathologic stage, n (%)			**0.044**
I	10 (12.5%)	3 (3.8%)	
II and III	28 (35%)	39 (48.8%)	
T stage, n (%)			**0.003**
T1	11 (13.8%)	2 (2.5%)	
T2	6 (7.5%)	3 (3.8%)	
T3	21 (26.2%)	34 (42.5%)	
T4	0 (0%)	3 (3.8%)	
N stage, n (%)			0.554
N0	11 (13.8%)	10 (12.5%)	
N1	10 (12.5%)	7 (8.8%)	
N2	7 (8.8%)	12 (15%)	
N3	10 (12.5%)	13 (16.2%)	
Histological type, n (%)			**< 0.001**
Diffuse Type	5 (6.2%)	23 (28.7%)	
Mucinous Type	1 (1.2%)	5 (6.2%)	
Papillary Type	7 (8.8%)	4 (5%)	
Signet Ring Type	8 (10%)	6 (7.5%)	
Tubular Type	17 (21.2%)	4 (5%)	
Histological grade, n (%)			**0.027**
G1 & G2	23 (28.7%)	14 (17.5%)	
G3	15 (18.8%)	28 (35%)	
Residual tumor, n (%)			0.776
R0	26 (32.5%)	31 (38.8%)	
R1 & R2	12 (15%)	11 (13.8%)	
Primary therapy outcome, n (%)			0.515
CR	25 (31.2%)	34 (42.5%)	
PD	8 (10%)	5 (6.2%)	
PR	2 (2.5%)	1 (1.2%)	
SD	3 (3.8%)	2 (2.5%)	
Anatomic neoplasm subdivision, n (%)			0.151
Antrum	10 (12.5%)	6 (7.5%)	
Cardia	15 (18.8%)	13 (16.2%)	
Fundus/Body	13 (16.2%)	20 (25%)	
other	0 (0%)	3 (3.8%)	
Age (years), meidan (IQR)	63 (57.25, 71.5)	66 (59, 70.75)	0.606

### High Expression of THBS2 Was Associated With Poor Prognosis of GC

**FIGURE 9:**
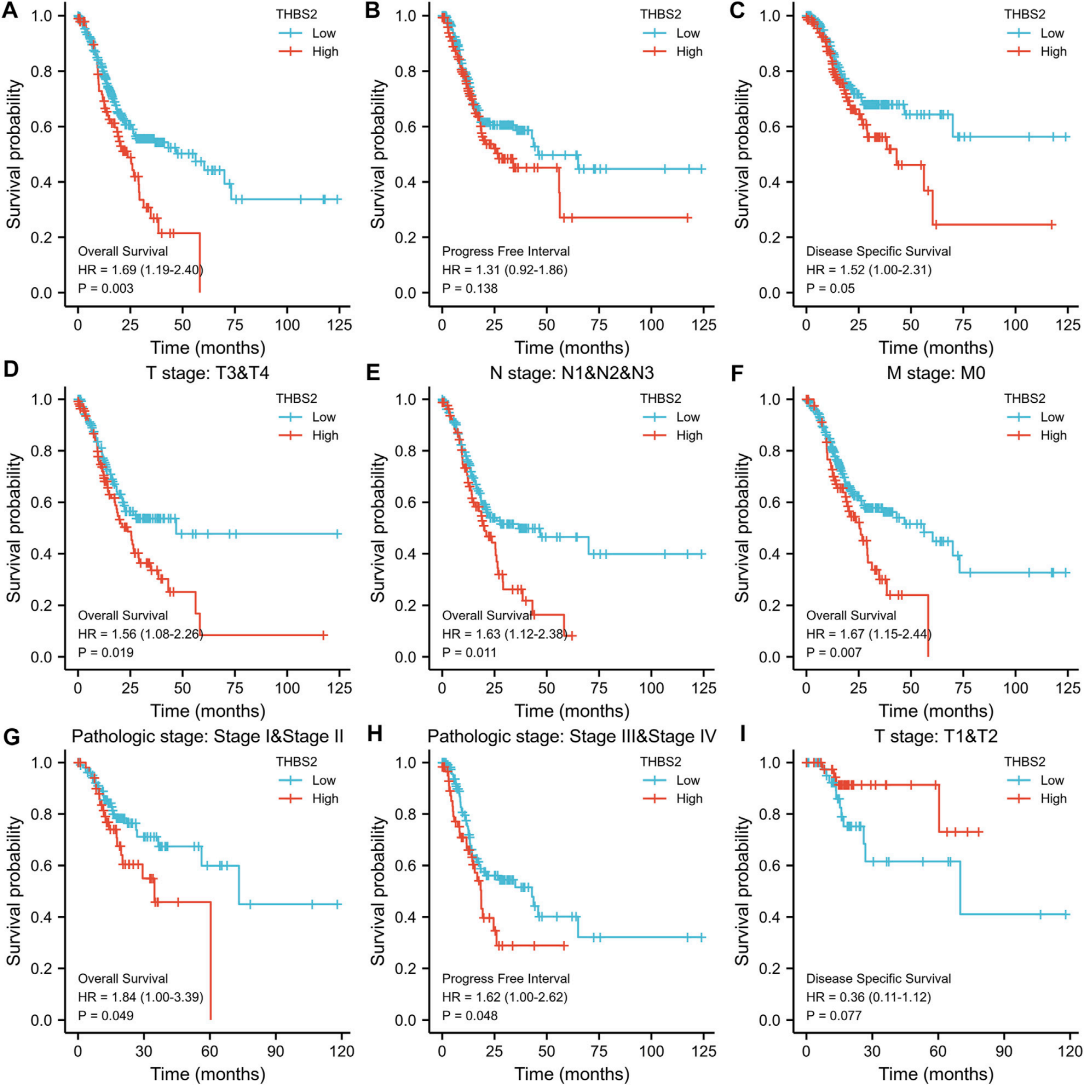
Kaplan-Meier survival curves comparing the high and low expression of THBS2 in GC. **(A–C)** Survival curves of OS, DSS, and PFI between THBS2-high and-low patients with GC. **(D–G)** OS survival curves of T3&T4, N1&N2&N3, M0, and stage IandII subgroups between THBS2-high and -low patients with GC. **(H)** PFI survival curves of stage IIIandIV subgroup between THBS2-high and -low patients with GC. **(I)** DSS survival curves of T1&T2 subgroup between THBS2-high and -low patients with GC. GC, gastric cancer; OS, overall survival; DSS, disease specific survival; PFI, progression free interval.

As can be seen from Figure 9A, the high THBS2 expression was significantly correlated with poor OS (*P* = 0.003). Moreover, high THBS2 expression was also associated with worse PFI and DSS; however, the difference was not statistically significant (Figures 9B,C).

The subgroup survival analysis showed that the prognosis of GC patients with THBS2 high expression was poor in T3 & T4 (HR = 1.56 [1.08–2.66]), N1 & N2 & N3 (HR = 1.63 [1.12–2.38]), M0 (HR = 1.67 [1.15–2.44]), and stage I and II (HR = 1.84 [1.00–3.39]) subgroups in OS, and stage III and IV (HR = 1.62 [1.00–2.26]) subgroup in PFI (Figures 9D–I). However, there was no significant difference among the subgroups of DSS.

A multivariate Cox regression analysis including age, primary therapy outcome, THBS2 expression level, pathologic stage, histologic grade, sex, race, and histological type was performed to eliminate the effect of confounders on univariate Cox regression analysis. Multivariate Cox regression analysis showed that age (>65 vs. ≤65 years, HR [95% CI] = 1.671 [1.127–2.476], *P* = 0.011), primary therapy outcome (CR vs. PD & SD & PR, HR [95% CI] = 4.715 [3.151–7.065]; *P* < 0.001), THBS2 (high vs. low, HR [95% CI] = 1.534 [1.037–2.270], *P* = 0.032), pathologic stage (stage I and II vs. III and IV, HR [95% CI] = 1.518 [1.008–2.285], *p* = 0.045), and histologic grade (G1 & G2 vs. G3, HR [95% CI] = 1.547 [1.019–2.348], *p* = 0.040) were significantly correlated with OS in patients with GC (Table 5). However, multivariate Cox regression indicated that THBS2 expression levels had no association with poor DSS and DSS PFI (Tables 6 and 7).

**TABLE 5:** Univariate and multivariate regression analysis of Overall Survival (OS) related factors in patients with GC.

Characteristics	Total(N)	Univariate analysis		Multivariate analysis	
		Hazard ratio (95% CI)	P Value	Hazard ratio (95% CI)	P Value
Age	367				
≤7 al	164	Reference			
>65	207	1.620 (1.154–2.276)	**0.005**	1.671 (1.127–2.476)	**0.011**
Primary.therapy.outcome	313				
CR	231	Reference			
PD&SD&PR	86	4.228 (2.905–6.152)	**<0.001**	4.715 (3.151–7.056)	**<0.001**
THBS2	370				
Low	188	Reference			
High	187	1.330 (0.956–1.851)	0.091	1.534 (1.037–2.270)	**0.032**
Pathologic.stage	347				
Stage I&Stage II	164	Reference			
Stage III&Stage IV	188	1.947 (1.358–2.793)	**<0.001**	1.518 (1.008–2.285)	**0.045**
Histologic.grade	361				
G1&G2	147	Reference			
G3	219	1.353 (0.957–1.914)	0.087	1.547 (1.019–2.348)	**0.040**
Gender	370				
Female	134	Reference			
Male	241	1.267 (0.891–1.804)	0.188		
Race	320				
White	238	Reference			
Asian&Black or African American	85	0.801 (0.515–1.247)	0.326		
Histological.type	132				
Diffuse Type	63	Reference			
Tubular Type	69	0.929 (0.534–1.614)	0.793		
Anatomic.neoplasm.subdivision	353				
Cardia/Proximal&Gastroesophageal Junction	89	Reference			
Antrum/Distal&Fundus/Body	268	0.919 (0.628–1.345)	0.663		

**TABLE 6:** Univariate and multivariate regression analysis of Disease Specific Survival (DSS) related factors in patients with GC.

Characteristics	Total(N)	Univariate analysis		Multivariate analysis	
		Hazard ratio (95% CI)	P Value	Hazard ratio (95% CI)	P Value
Age	346				
≤6 al	164	Reference			
>65	207	1.211 (0.797–1.840)	0.371		
Primary.therapy.outcome	310				
CR	231	Reference			
PD&SD&PR	86	8.697 (5.439–13.908)	**<0.001**	8.590 (5.270–14.000)	**<0.001**
THBS2	349				
Low	188	Reference			
High	187	1.195 (0.786–1.815)	0.405		
Pathologic.stage	331				
Stage I&Stage II	164	Reference			
Stage III&Stage IV	188	2.146 (1.352–3.404)	**0.001**	1.439 (0.896–2.312)	0.132
Histologic.grade	340				
G1&G2	147	Reference			
G3	219	1.338 (0.862–2.078)	0.194		
Gender	349				
Female	134	Reference			
Male	241	1.573 (0.985–2.514)	0.058	1.228 (0.753–2.002)	0.410
Race	305				
White	238	Reference			
Asian&Black or African American	85	1.097 (0.656–1.836)	0.724		
Histological.type	129				
Diffuse Type	63	Reference			
Tubular Type	69	0.897 (0.480–1.676)	0.734		
Anatomic.neoplasm.subdivision	337				
Cardia/Proximal&Gastroesophageal Junction	89	Reference			
Antrum/Distal&Fundus/Body	268	0.767 (0.485–1.215)	0.259		

**TABLE 7:** Univariate and multivariate regression analysis of Progress Free Interval (PFI) related factors in patients with GC.

Characteristics	Total(N)	Univariate analysis		Multivariate analysis	
		Hazard ratio (95% CI)	P Value	Hazard ratio (95% CI)	P Value
Age	369				
≤9 al	164	Reference			
>65	207	0.858 (0.603–1.221)	0.395		
Primary.therapy.outcome	315				
CR	231	Reference			
PD&SD&PR	86	8.041 (5.465–11.832)	**<0.001**	8.704 (5.733–13.215)	**<0.001**
THBS2	372				
Low	188	Reference			
High	187	1.149 (0.807–1.637)	0.441		
Pathologic.stage	349				
Stage I&Stage II	164	Reference			
Stage III&Stage IV	188	1.676 (1.154–2.435)	**0.007**	1.037 (0.696–1.544)	0.858
Histologic.grade	363				
G1&G2	147	Reference			
G3	219	1.540 (1.057–2.245)	**0.025**	1.799 (1.186–2.730)	**0.006**
Gender	372				
Female	134	Reference			
Male	241	1.638 (1.099–2.440)	**0.015**	1.361 (0.893–2.077)	0.152
Race	322				
White	238	Reference			
Asian&Black or African American	85	1.061 (0.688–1.637)	0.787		
Histological.type	132				
Diffuse Type	63	Reference			
Tubular Type	69	0.806 (0.466–1.392)	0.438		
Anatomic.neoplasm.subdivision	354				
Cardia/Proximal&Gastroesophageal Junction	89	Reference			
Antrum/Distal&Fundus/Body	268	0.696 (0.474–1.022)	0.064	0.802 (0.522–1.234)	0.316

### Construction and Validation of Nomogram

**FIGURE 10:**
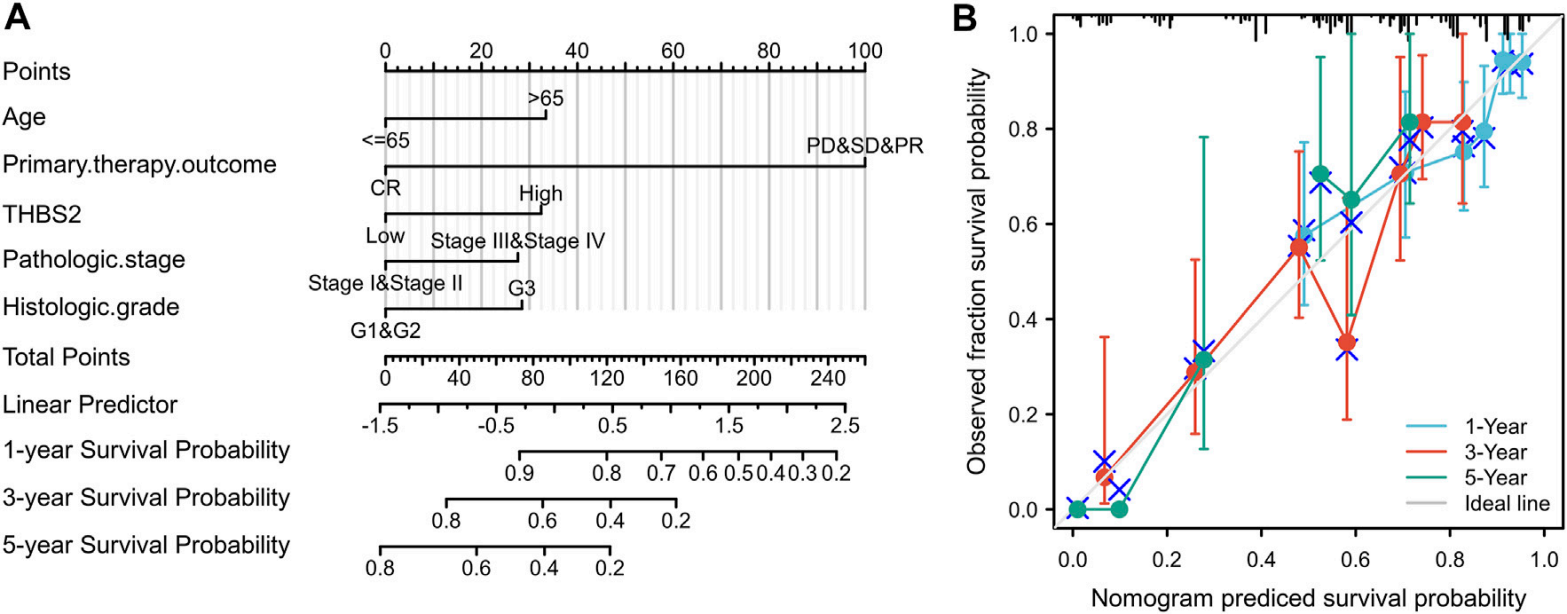
A nomogram and calibration plot to predict GC patients’ 1-, 3-, and 5-years OS. **(A)** A nomogram for predicting the 1-, 3-, and 5-years OS for GC patients. **(B)** Calibration plots of the nomogram. GC, gastric cancer; OS, overall survival.

All parameters persisting as independent predictors in the multivariate Cox models of the subgroups, including age, primary therapy outcome, THBS2 expression, pathological stage, and histologic grade, were integrated into the nomogram (Figure 10). The nomogram and calibration blots demonstrated that the nomogram-predicted 1-, 3-, and 5-years survival probabilities of GC were similar to the actual probabilities (C-index [95%CI] = 0.725 [0.701–0.750]), indicating that the prediction was in good agreement with the actual survival probability of GC (Figure 10B). These results indicated that the prediction ability of the nomogram for the survival probabilities might be clinically applicable also suggests that the nomogram was well-calibrated, with the mean predicted probabilities close to observed probabilities.

## Discussion

By analyzing the expression profile of GC patients in the open online database, we found that the expression level of THBS2 in GC tissues was significantly higher than that in cancer-adjacent tissues. There was also a significant difference in THBS2 expression in GC tissues and corresponding adjacent tissues, which was validated using RT-PCR and IHC. Additionally, the ROC curve demonstrated that THBS2 had a potential predictive value in distinguishing GC from cancer-adjacent tissues (AUC = 0.864, 95% CI: 0.812–0.915). This observation suggests that THBS2 may serve as a potential indicator for GC diagnosis.

Totally, 599 DEGs based on THBS2 expression levels were screened, including 170 up-regulated and 429 down-regulated DEGs. The results of GO enrichment analysis showed that the enrichment was primarily related to collagen and extracellular matrix (ECM). In the course of cancer progression, there are apparent changes in ECM. Collagen together with laminin and fibronectin constitutes a key substrate for the growth and migration of cancer cells as a major component of the ECM (Bonnans et al., 2014). Similarly, studies have also demonstrated that the ECM is an important component of all cancer markers that play a crucial role (Andreuzzi et al., 2020). ECM receptor interactions are critical in the tumor microenvironment (Chen et al., 2021), tumorigenesis, and tumor progression (Malik et al., 2015). The ECM protein regulates the metastasis of GC cells through the ITGB4/FAK/SOX2/HIF-1α signaling pathway induced by ECM receptor interaction (Gan et al., 2018). For KEGG pathway enrichment analysis, the DEGs are enriched in ECM-receptor interaction during S. aureus infection. The structural function of ECM is critical to maintaining normal cell activity (Giussani et al., 2019). ECM promotes tumorigenesis and progression by avoiding apoptosis, regulating cell growth, promoting tumor angiogenesis, and acquiring the ability of invasion and metastasis (Pickup et al., 2014; Malik et al., 2015; Poltavets et al., 2018; Eble and Niland, 2019). Signaling pathways associated with protein digestion and absorption are also critical for tumor progression (Mo et al., 2020). Therefore, treatment targeting ECM and tumor angiogenesis may be effective in preventing metastasis and recurrence of GC (Gan et al., 2018). Additionally, GSEA analysis demonstrated that THBS2-related enrichment pathways were as follows: focal-adhesion, VEGF signaling, Wnt signaling, cytokine-cytokine-receptor interaction, immunoregulatory lymphoid and a non-lymphoid cell, senescence, and autophagy in cancer. These pathways are closely related to cell adhesion, immune regulation, tumor autophagy, tumor progression, and tumor angiogenesis, respectively. Adhesion to ECM via specific focal adhesion points is an important step in cancer cell migration and invasion (Paluch et al., 2016; Ringer et al., 2017). VGFR related signaling pathway is important in the molecular pathogenesis of tumor growth and metastasis (Adamis and Shima, 2005). THBS2 may play a crucial role in the occurrence and progression of GC, so it is reasonable to speculate that THBS2 may be a potential therapeutic target for GC.

Previous studies have confirmed that the interaction between tumor-infiltrating immune and malignant cells leads to the immune invasion of tumors, as the immune system plays a dual role by supporting both tumor progression and host defense (Bremnes et al., 2011). This study showed that the abundance of macrophages and NK cells in the tumor microenvironment of GC with high THBS2 expression was higher than that of GC with low THBS2 expression, while the abundance of Th17 cells was lower. Single tumor-initiating cells were detected to recruit polarized M2-like macrophages and assist evasion from immune clearance (Guo et al., 2017). Moreover, macrophages can promote tumor invasion and metastasis (Doak et al., 2018). In some tumor types such as ovarian, prostate, and colorectal cancer, Th17 cells induce an antitumor immune response by recruiting cytotoxic effector T cells and producing effector cytokines, including interferon-gamma (Kryczek et al., 2009; Tosolini et al., 2011). Therefore, we speculated that the high expression of THBS2 might affect the disease progression and prognosis of patients with GC by inducing changes in the tumor microenvironment.

THBS2, a member of the thrombospondin family of multidomain and secreted multicellular calcium-binding glycoproteins, mediates cell-to-cell and cell-to-matrix interactions (Ng et al., 2021). The expression level of THBS2 in colon cancer was significantly increased, and the higher the expression level of THBS2, the worse the OS of patients (Wang et al., 2016b). The high expression of THBS2 promotes the metastasis of colon cancer and is associated with an advanced clinical stage (Wang et al., 2016b). Our results suggest that the high expression of THBS2 was significantly correlated with the pathological grade (stage I vs. stage II and III & IV, *P* < 0.01), histological grade (G1 & G2 vs. G3, *p* < 0.05), histological type (Diffuse Type vs. Tubular Type, *P* < 0.05), T stage (T1 vs. T2, T1 vs. T3, T1 vs. T4, *P* < 0.001) of patients. The high THBS2 expression was significantly correlated with poor OS. Additionally, the subgroup survival analysis demonstrated that the prognosis of GC patients with THBS2 high expression was poor in T3 & T4, N1 & N2 & N3, M0, and stage I and II subgroups in OS, and stage III and IV subgroups in PFI. This study also indicates that the high expression of THBS2 may accelerate GC progression, leading to a poor prognosis. The higher the expression level of THBS2, the worse the degree of differentiation of GC.

Multivariate Cox regression analysis demonstrated that age, primary therapy outcome, THBS2 expression, pathological stage, and histologic grade were independent risk factors associated with poor OS in GC. A nomogram based on the above independent risk factors affecting OS of GC was also established to predict the survival probability of patients with GC. The survival probability of GC patients predicted by the constructed nomogram was similar to the actual survival probability, indicating that the created nomogram could be successfully used to predict the survival of GC patients. The accurate prediction of survival of cancer patients can provide essential information for individualized treatment of cancer patients (Erickson et al., 2014; Gratian et al., 2014). Our nomogram to predict the survival probability of GC has been verified using a calibration blot and should be clinically promoted.

## Conclusion

Overall, the findings of the current research are summarized below:

Firstly, the THBS2 expression level in GC was significantly higher than that in para-cancer tissues, and THBS2 may be a potential biomarker for GC diagnosis. Secondly, THBS2 may affect the progression and prognosis of GC by changing the tumor microenvironment and may be a potential therapeutic target for GC. Thirdly, histologic grade, primary therapy outcome, age, and THBS2 might be independent risk factors associated with poor OS in GC. Finally, the nomogram may provide more individualized prognostic information for patients with GC. However, neither the TCGA data nor the hospital data collected were sufficiently large. Therefore, more information should be collected to verify the accuracy of the results.

## Data Availability

Publicly available datasets were analyzed in this study. This data can be found here: https://portal.gdc.cancer.gov/ http://xena.ucsc.edu/&lt;/b&gt.
